# Topical cutaneous application of CO_2_ accelerates bone healing in a rat femoral defect model

**DOI:** 10.1186/s12891-019-2601-5

**Published:** 2019-05-22

**Authors:** Yu Kuroiwa, Tomoaki Fukui, Shunsuke Takahara, Sang Yang Lee, Keisuke Oe, Michio Arakura, Yohei Kumabe, Takahiro Oda, Tomoyuki Matsumoto, Takehiko Matsushita, Toshihiro Akisue, Yoshitada Sakai, Ryosuke Kuroda, Takahiro Niikura

**Affiliations:** 1 Department of Orthopaedic Surgery, Kobe University Graduate School of Medicine, 7-5-1 Kusunoki-cho, Chuo-ku, Kobe, 650-0017 Japan; 2 Department of Orthopaedic Surgery, Hyogo Prefectural Kakogawa Medical Center, 203 Kanno, Kanno-cho, Kakogawa, 675-8555 Japan; 3 Department of Orthopaedic Surgery, Showa University School of Medicine, 1-5-8 Hatanodai, Shinagawa-ku, Tokyo, 142-8666 Japan; 4 Department of Rehabilitation Science, Kobe University Graduate School of Health Sciences, 7-10-2 Tomogaoka, Suma-ku, Kobe, 654-0142 Japan; 5 Division of Rehabilitation Medicine, Kobe University Graduate School of Medicine, 7-5-1 Kusunoki-cho, Chuo-ku, Kobe, 650-0017 Japan

**Keywords:** CO_2_, Bone healing, Bone defect

## Abstract

**Background:**

Bone defects may occur because of severe trauma, nonunion, infection, or tumor resection. However, treatments for bone defects are often difficult and have not been fully established yet. We previously designed an efficient system of topical cutaneous application of carbon dioxide (CO_2_) using a novel hydrogel, which facilitates CO_2_ absorption through the skin into the deep area within a limb. In this study, the effect of topical cutaneous application of CO_2_ on bone healing was investigated using a rat femoral defect model.

**Methods:**

In this basic research study, an in vivo bone defect model, fixed with an external fixator, was created using a rat femur. The affected limb was shaved, and CO_2_ was applied for 20 min/day, 5 days/week. In the control animals, CO_2_ gas was replaced with air. Radiographic, histological, biomechanical, and genetic assessments were performed to evaluate bone healing.

**Results:**

Radiographically, bone healing rate was significantly higher in the CO_2_ group than in the control group at 4 weeks (18.2% vs. 72.7%). The degree of bone healing scored using the histopathological Allen grading system was significantly higher in the CO_2_ group than in the control group at 2 weeks (1.389 ± 0.334 vs. 1.944 ± 0.375). The ultimate stress, extrinsic stiffness, and failure energy were significantly greater in the CO_2_ group than in the control group at 4 weeks (3.2 ± 0.8% vs. 38.1 ± 4.8%, 0.6 ± 0.3% vs. 41.5 ± 12.2%, 2.6 ± 0.8% vs. 24.7 ± 5.9%, respectively.). The volumetric bone mineral density of the callus in micro-computed tomography analysis was significantly higher in the CO_2_ group than in the control group at 4 weeks (180.9 ± 43.0 mg/cm^3^ vs. 247.9 ± 49.9 mg/cm^3^). Gene expression of vascular endothelial growth factor in the CO_2_ group was significantly greater than that in the control group at 3 weeks (0.617 ± 0.240 vs. 2.213 ± 0.387).

**Conclusions:**

Topical cutaneous application of CO_2_ accelerated bone healing in a rat femoral defect model. CO_2_ application can be a novel and useful therapy for accelerating bone healing in bone defects; further research on its efficacy in humans is warranted.

## Background

Bone defects can occur during and after treatments for bone tumor, osteomyelitis, and severe trauma, and they often lead to significant problems in the clinical setting. Treatments for these bone defects usually require external fixation procedures or microsurgical techniques, including ascension of a corticotomized bone fragment with an Ilizarov fixator [1], a free vascularized fibular flap [2, 3], an osteomyocutaneous flap [4], and Masquelet’s two-stage technique [5]. However, these clinically applicable therapies for bone defects are cumbersome and time-consuming, and treatment options are limited. Hence, basic and clinical research on bone healing that could lead to an improvement in current treatments for bone defects are urgently required.

These days, topical cutaneous application of CO_2_ is drawing attention in various fields, such as health, medical care, beauty, and sports [6–13]. These therapeutic effects are caused by an increase in the blood flow, microcirculation, and nitric-oxide-dependent neocapillary formation, as well as by an increase in the oxygen partial pressure in the local area known as the Bohr effect [10–14]. However, the detailed mechanism of these effects is still unknown.

We previously designed a novel system of topical cutaneous CO_2_ application by means of 100% CO_2_ gas and a hydrogel, which can facilitate CO_2_ absorption through the skin into the deep area within a limb [15, 16]. This easy and noninvasive system increased blood flow and oxygen dissociation from hemoglobin in the soft tissues surrounding a bone by way of an artificial Bohr effect [15]. Moreover, it was demonstrated that CO_2_ application significantly increased the gene expression of vascular endothelial growth factor (VEGF) in the rat muscle [16]. The effect of CO_2_ application in accelerating fracture repair via promoting angiogenesis, blood flow, and endochondral ossification was also demonstrated in a rat femoral fracture model [17]. A similar effect to that seen in a rat fracture model can be expected to be effective for bone defects as well. However, to date, the effect of topical cutaneous application of CO_2_ on bone defects has not been studied yet.

Therefore, in this study, we hypothesized that topical cutaneous application of CO_2_ accelerates bone healing and investigated the effect of CO_2_ application on bone healing using a rat femoral defect model.

## Methods

### Animal model

**Fig. 1:**
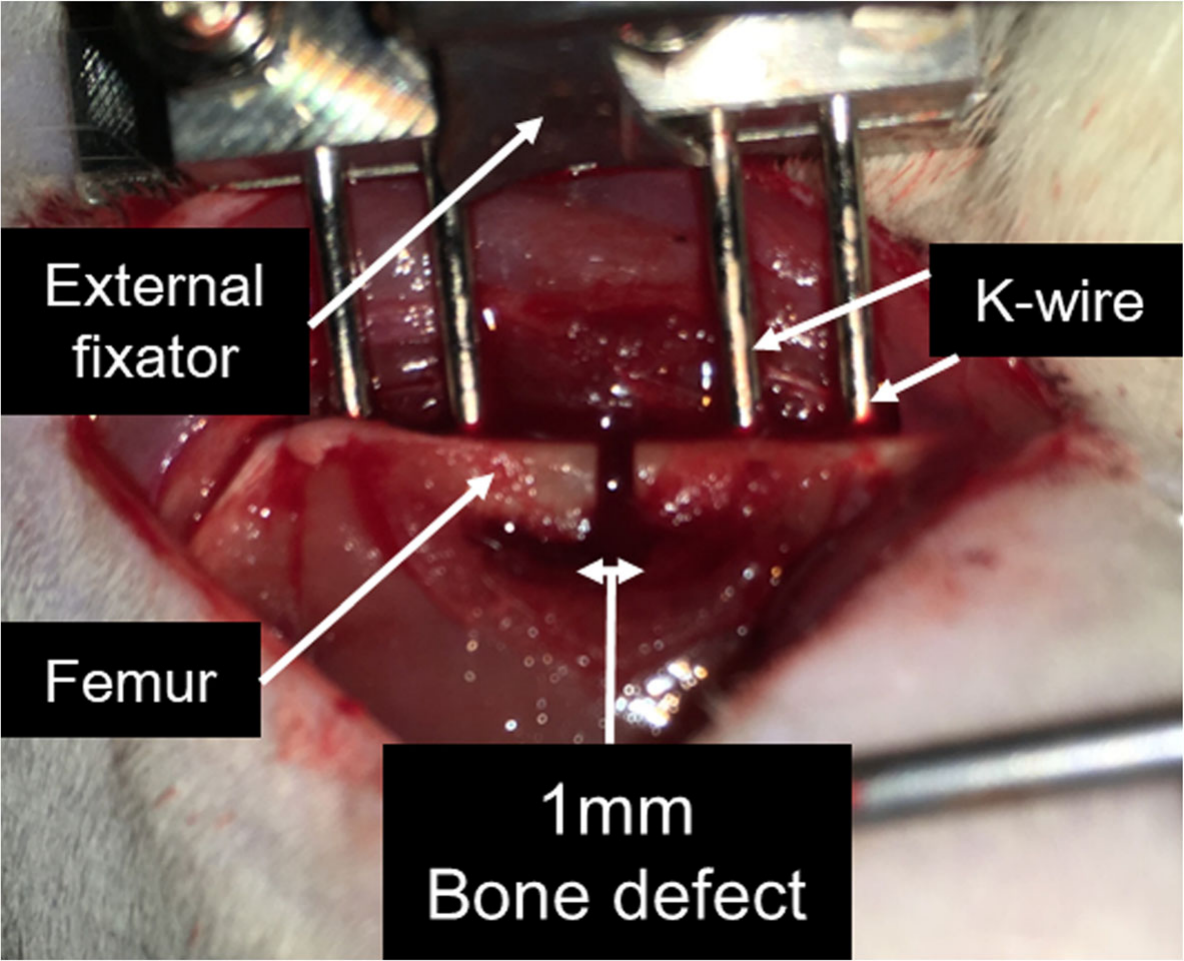
Representative intraoperative image. After exposing the femoral shaft, two pairs of tip-threaded 1.4-mm-diameter K-wires were placed in the proximal and distal femur. A bone defect of 1 mm was created between the proximal and distal pins by an oscillating saw, and a custom-made external fixator was attached to connect the pairs of pins

A total of 92 male Fischer 344 rats (Japan SLC, Hamamatsu, Japan) were used, with approval from the institutional ethical committee of our institute. Surgeries were performed on all animals at age 12 weeks under general anesthesia with isoflurane and pentobarbital. After exposing the femoral shaft, two pairs of tip-threaded 1.4-mm-diameter K-wires were placed in the proximal and distal femur. A bone defect of 1 mm was created between the proximal and distal pins by an oscillating saw, and a custom-made external fixator was attached to connect the pairs of pins (Fig. 1). Euthanasia was performed by intraperitoneal administration of a pentobarbital overdose before assessment.

### Topical cutaneous application of CO_2_

**Fig. 2:**
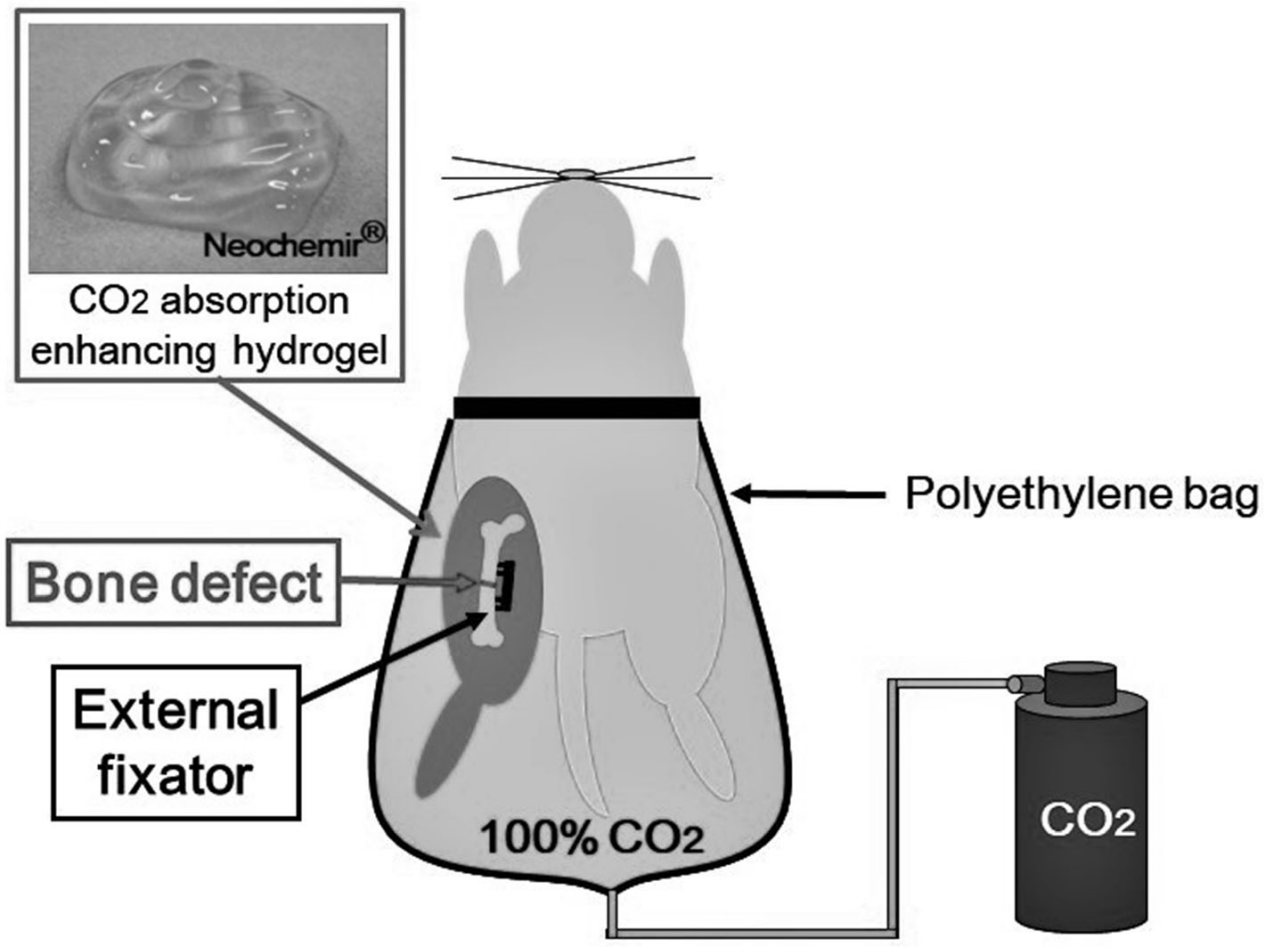
An illustration of the method of CO_2_ application in a rat femoral defect model. The affected limb was sealed in a polyethylene bag filled with 100% CO_2_ for 20 min a day

Rats were assigned into two groups: CO_2_ group (*n* = 46) and control group (n = 46). After sedation using a minimum dose of isoflurane, the hair of the affected limb was shaved and CO_2_ absorption enhancing hydrogel (NeoChemir Inc., Kobe, Japan) was applied. The hydrogel had a pH of 5.5 and consisted of carbomer, glycerin, sodium hydroxide, sodium alginate, sodium dihydrogen phosphate, methylparaben, and deionized water. In the CO_2_ group, the affected limb was sealed in a polyethylene bag filled with 100% CO_2_ for 20 min a day (Fig. 2). This treatment was performed 5 days a week. In the control group, the affected limb, coated in the same way with CO_2_ absorption enhancing hydrogel, was sealed in a polyethylene bag filled with air.

### Radiographic assessment of bone healing

At 1, 2, 3, and 4 weeks after surgery, 58 animals (29 in the CO_2_ group and 29 in the control group) were anesthetized and fixed in the supine position with the hip joints fully abducted, and radiographs of the defect site were acquired. Radiographically, each callus on the two cortices on the lateral images were evaluated by three orthopedic surgeons blinded to the groups. Bone healing was defined as the absence of a bony gap or the presence of a bridging callus at both the anterior and posterior cortices.

### Histological assessment for bone healing

At 2 and 4 weeks after surgery, the femur was harvested from 6 animals in each group, and the tissues were fixed in 4% paraformaldehyde, decalcified with ethylenediaminetetraacetic acid (EDTA), and embedded in paraffin wax. Sagittal sections were cut to a thickness of 5 μm and stained with Safranin-O/Fast Green for histological assessment of the bone defect area. The degree of bone healing was assessed with Allen’s grading system (grades 0 through 4), which was originally designed for histological evaluation of fracture healing [18]. Specimens were examined by three blinded examiners.

### Biomechanical assessment of bone healing

Five femurs at 4 weeks after surgery were used for biomechanical evaluation. After euthanasia, the femur was dissected, and the muscle surrounding the defect site was removed. A standardized three-point bending test was performed with a load torsion and bending tester (MZ-500D, MZ-500S, Maruto Instrument Co., Ltd., Tokyo, Japan) for rats in both groups. The ultimate stress (N), extrinsic stiffness (N/mm), and failure energy (N.mm) were assessed by outsourcing to Kureha Special Laboratory, Co., Ltd. (Tokyo, Japan).

### Micro-computed tomography (μ-CT) measurement of bone healing

At 4 weeks after surgery, the femur with bone defect was harvested, and the external fixator and pins were removed from the bone. For quantification of bone regeneration, μ-CT imaging analysis was performed. The femurs were scanned and evaluated using a μ-CT scanner (R_mCT2, RIGAKU, Tokyo, Japan). The region of interest (ROI) was set as 3 mm proximal and distal from the midline of the defect site on sagittal view. Tissue mineral density (TMD), total callus volume (TV), bone mineral content (BMC), and volumetric bone mineral density (vBMD; BMC/TV) of the callus were evaluated by TRI/3-D-BON (Ratoc System Engineering, Tokyo, Japan).

### Assessment of gene expression

At 1, 2, and 3 weeks after surgery, gene expression was measured in 6 animals in each group by real-time polymerase chain reaction (PCR). Newly generated callus tissue was harvested. Total RNA was extracted from the tissue with a RNeasy Mini Kit (Qiagen, Valencia, California) and reverse-transcribed into single-stranded DNA with a high-capacity cDNA reverse transcription kit (Applied Biosystems, Foster City, California). Real-time PCR was performed in duplicate on the cDNA with an ABI PRISM 7700 Sequence Detection System and SYBR Green reagent (Applied Biosystems). We examined the expression of genes for VEGF to evaluate angiogenesis. The expression level of each gene was first normalized with respect to that of glyceraldehyde-3-phosphate dehydrogenase (GAPDH), which served as an internal control, and the results are presented as the fold change relative to the control group (ΔΔCt method).

### Statistical analysis

The Fisher’s exact test was used for radiographic assessment and the Mann-Whitney U-test was used for histological assessment, μ-CT assessment, biomechanical assessment, and genetic assessment with real-time PCR. A *p*-value less than 0.05 was considered statistically significant.

## Results

### Radiographic assessment of bone healing

**Fig. 3:**
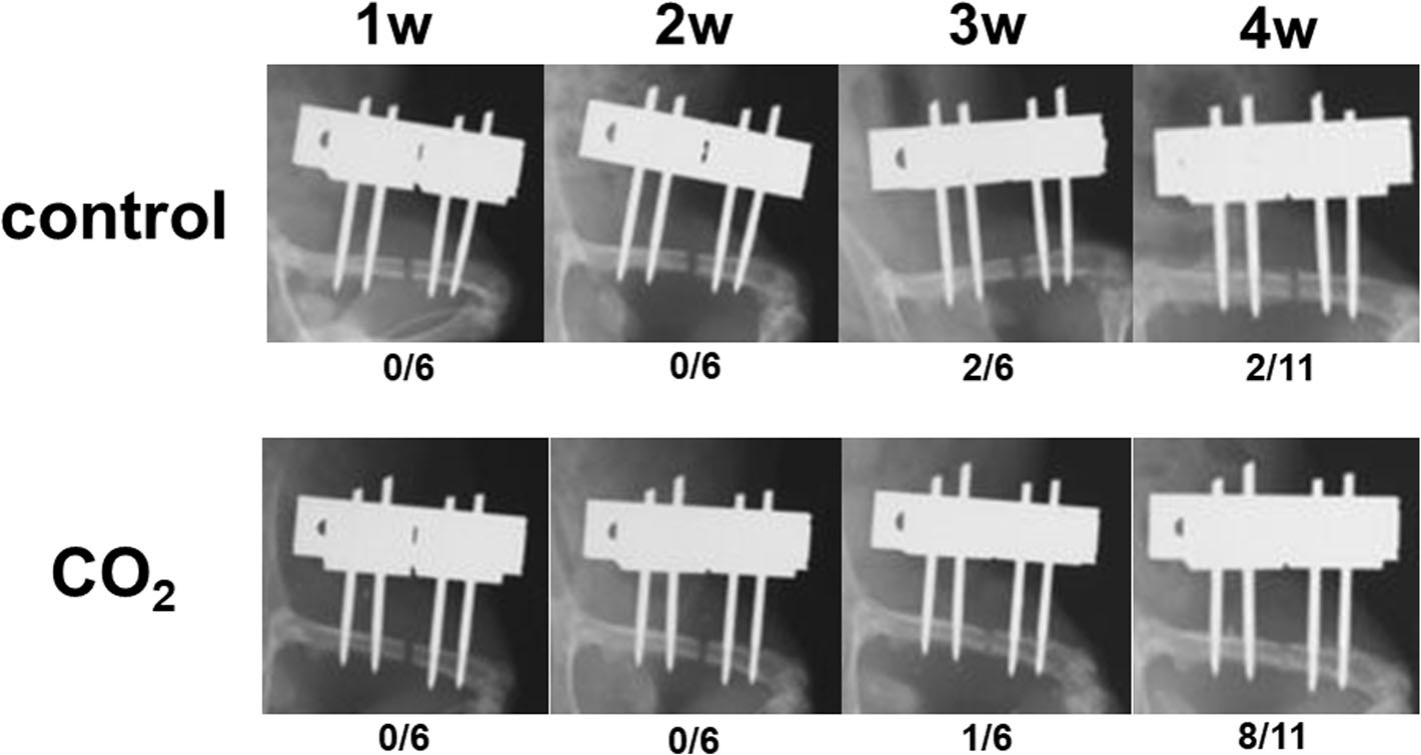
Radiographic evidence of bone healing in each group at 1, 2, 3, and 4 weeks after surgery. The number of samples with bone healing is represented under the radiograph

**Fig. 4:**
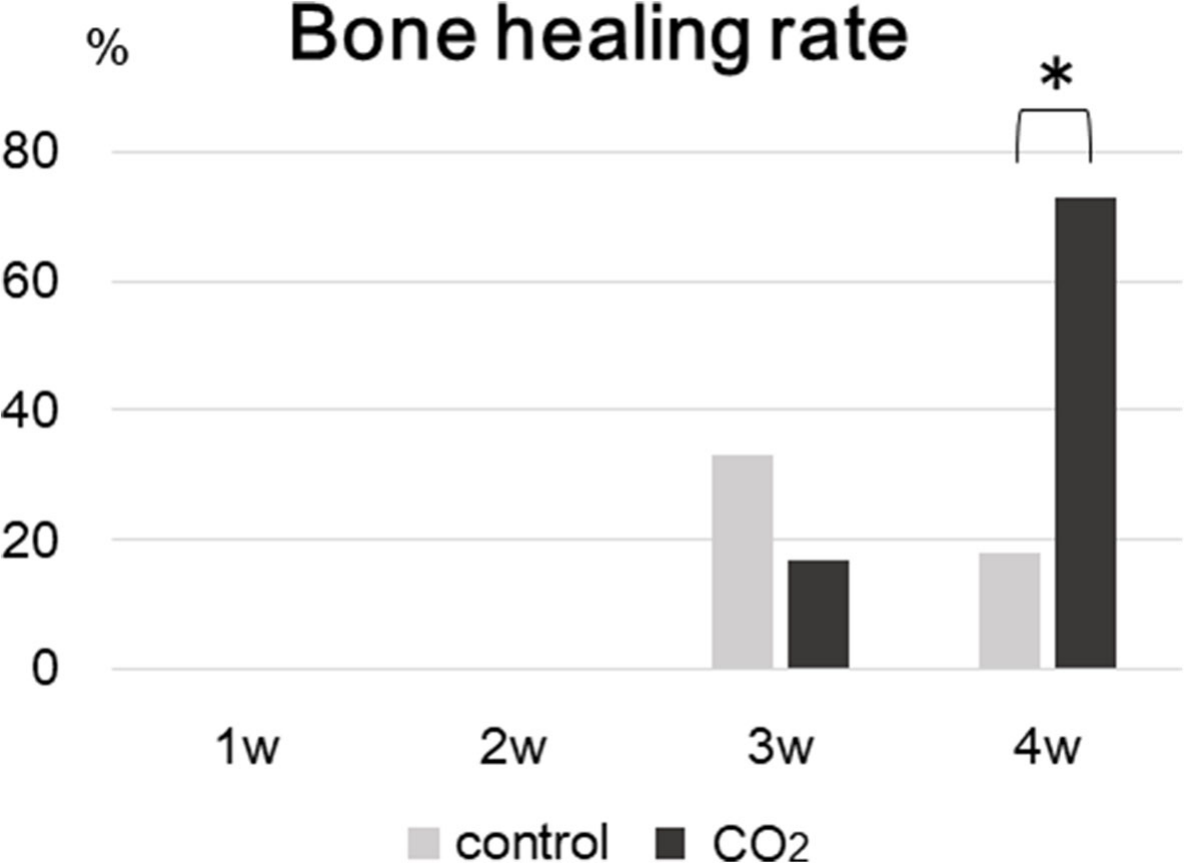
Bone healing rates in each group determined by radiographic assessment at 1, 2, 3, and 4 weeks. There was a significant difference in the bone healing rate between the two groups at 4 weeks. (*p < 0.05 in the indicated group)

Representative radiographic findings of the control and CO_2_ groups at 1, 2, 3, and 4 weeks are shown in Fig. 3. At 4 weeks, 8 rats (72.7%) in the CO_2_ group achieved bone healing, whereas the femurs of 2 rats (18.2%) showed bone healing in the control group (Table 1). There was a significant difference in the bone healing rate between the two groups. (Fig. 4) However, there was no significant difference in the bone healing rate between both groups at 1, 2, and 3 weeks.

**Table 1:** Healing and non-healing rates determined by radiographic assessment at 4 weeks

	Control	CO_2_
Healing	2/11 (18.2%)	8/11 (72.7%)*
Non-healing	9/11 (81.8%)	3/11 (27.3%)

Bone healing was achieved in 8 rats (72.7%) in the CO_2_ group, whereas the femurs of 2 rats (18.2%) showed bone healing in the control group. There was a significant difference in the bone healing rate between the two groups.* *p* < 0.05 compared to the control group

### Histological assessment for bone healing

**Fig. 5:**
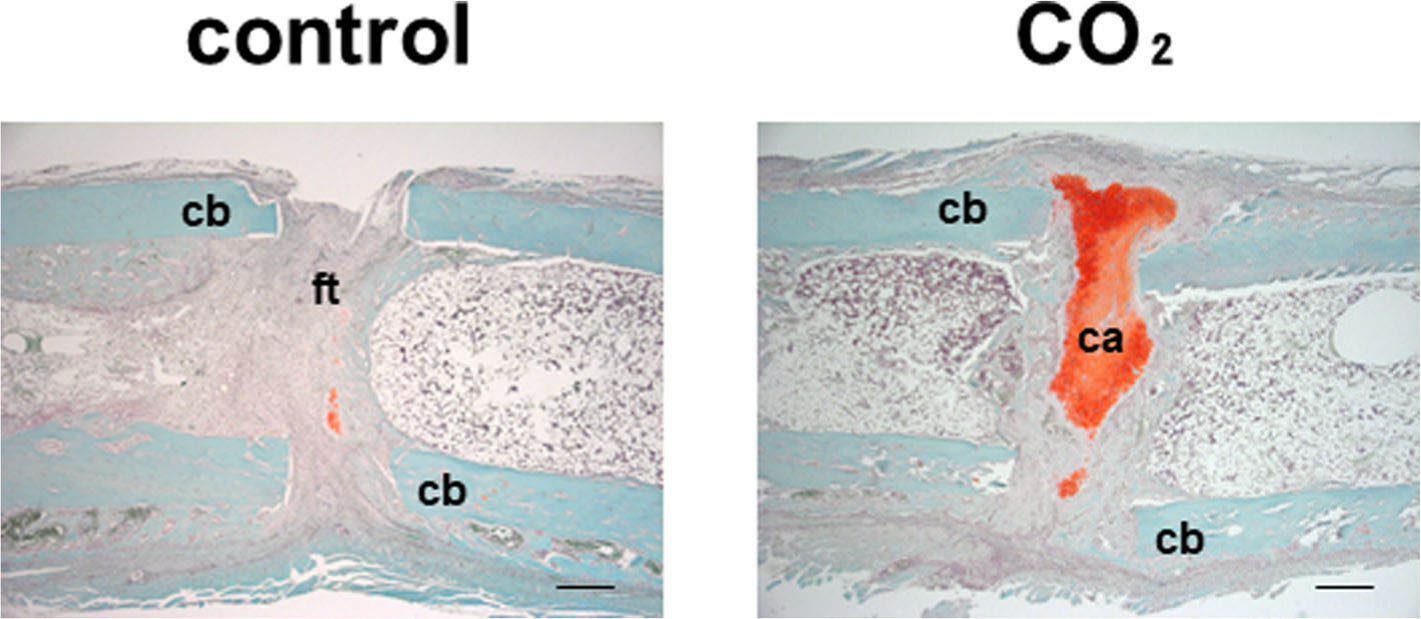
Representative histological sections stained with Safranin-O/Fast Green. cb = cortical bone, ca = cartilage, and ft. = fibrous tissue. Bar = 500 μm

**Fig. 6:**
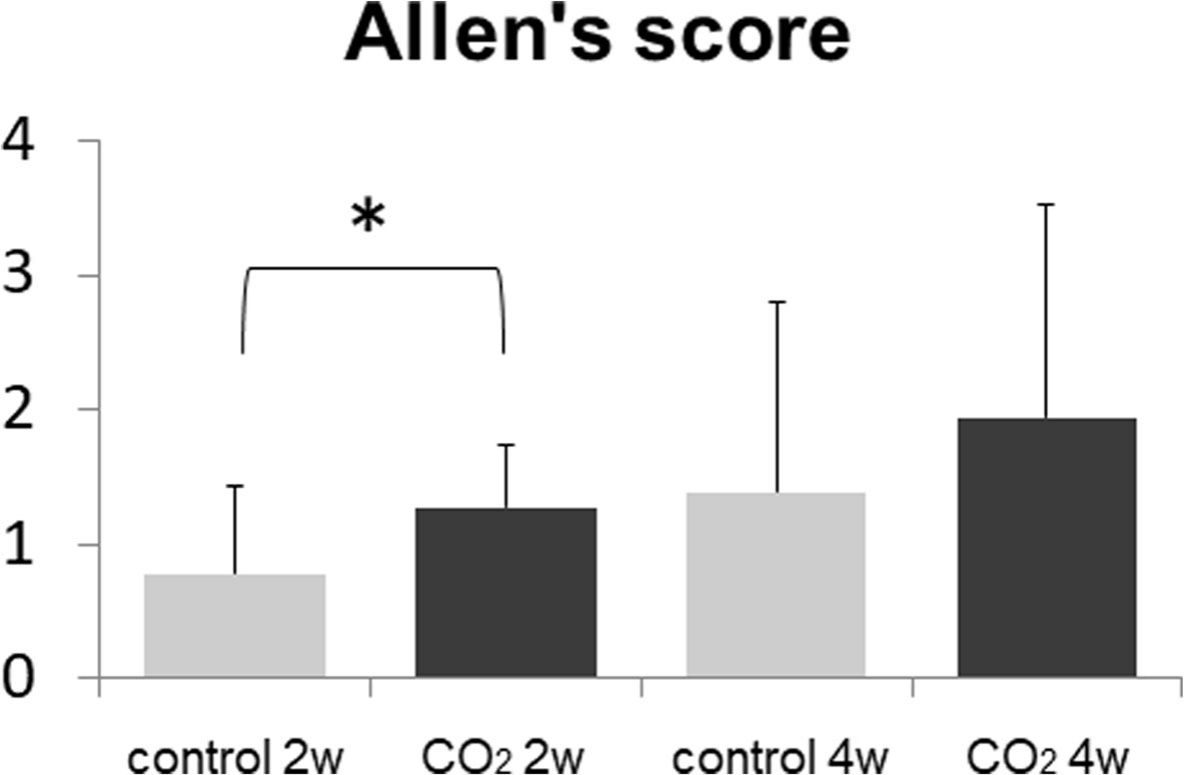
The degree of bone healing as indicated by the mean Allen’s score at 2 and 4 weeks. (*n* = 6 in each group) (*p < 0.05 in the indicated group)

The representative histological findings of control and CO_2_ groups at 2 weeks are shown in Fig. 5. The area of cartilage stained red with Safranin-O was larger in the CO_2_ group than in the control group, which suggested that the degree of bone healing via endochondral ossification was more accelerated in the CO_2_ group than in the control group. The evaluation of histological bone healing with Allen’s grading score is shown in Fig. 6. A significant difference between the CO_2_ and control groups was detected at 2 weeks, but no significant difference was observed in the degree of bone healing between both groups at 4 weeks.

### Biomechanical assessment of bone healing

**Fig. 7:**
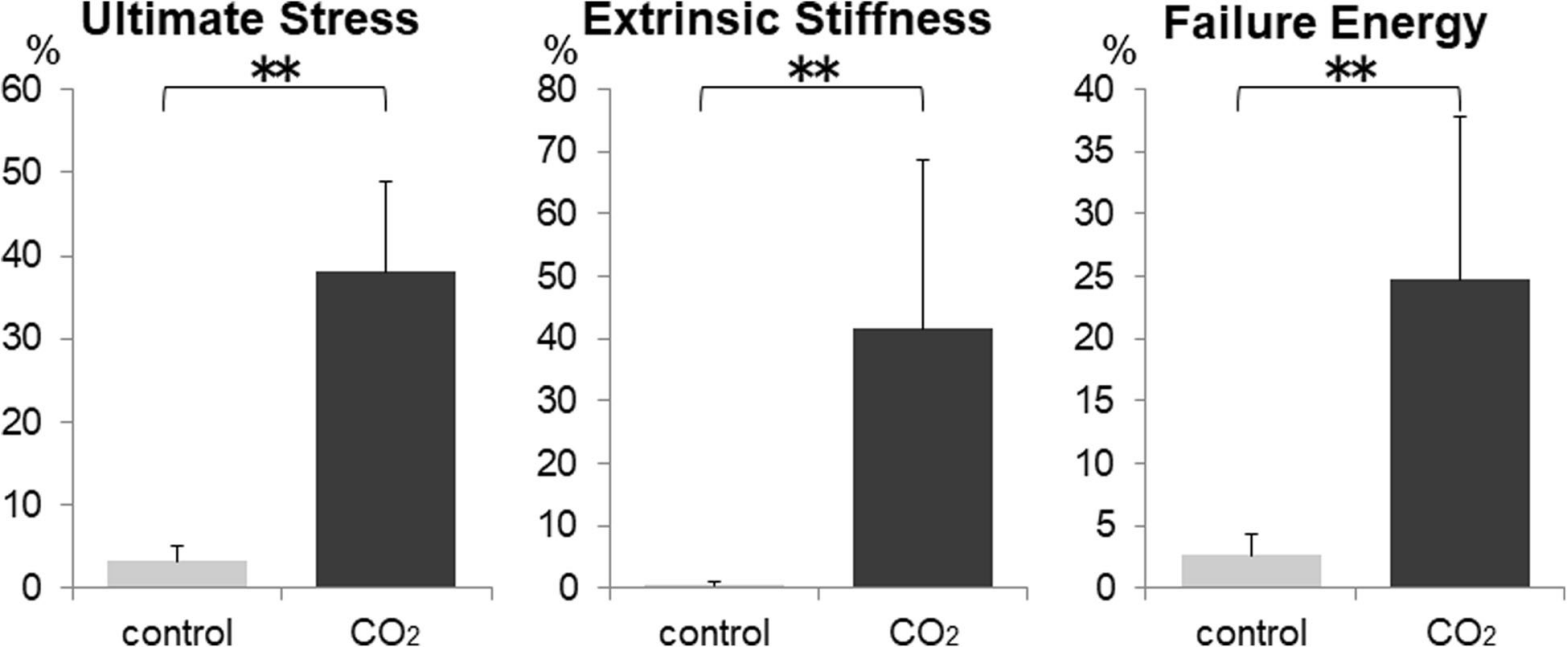
Biomechanical assessment of a femoral defect as assessed by the three-point bending test at 4 weeks. (*n* = 5 in each group) Values were normalized relative to the contralateral, intact femur. (**p < 0.01 in the indicated groups)

The ultimate stress, extrinsic stiffness, and failure energy of the defected femurs, that were expressed as a percentage of the value in the intact femur, were significantly higher in the CO_2_ group than those in the control group (3.2% vs. 38.1, 0.6% vs. 41.5, 2.6% vs. 24.7%, respectively.) (Fig. 7).

### Micro-computed tomography (μ-CT) measurement of bone regeneration

A morphometrical assessment was performed with μ-CT at 4 weeks after surgery. The vBMD of the callus was significantly greater in the CO_2_ group than in the control group. No significant differences in TMD, TV, and BMC were observed between the control and CO_2_ groups. (Table 2).

**Table 2:** Micro-computed tomography (μ-CT) measurement at 4 weeks

Parameter	Control	CO_2_
TMD (mg/cm^3^)	605.6 ± 55.5	635.8 ± 55.2
TV (cm^3^)	0.062 ± 0.0097	0.063 ± 0.014
BMC (mg)	11.2 ± 2.83	15.7 ± 4.68
vBMD (mg/cm^3^)	180.9 ± 43.0	247.9 ± 49.9*

The vBMD of the callus was significantly greater in the CO_2_ group than in the control group. * *p* < 0.05 compared to the control group. *TMD* tissue mineral density, *TV* total callus volume; *BMC* bone mineral content; *vBMD* volumetric bone mineral density (BMC/TV)

### Assessment of gene expression

**Fig. 8:**
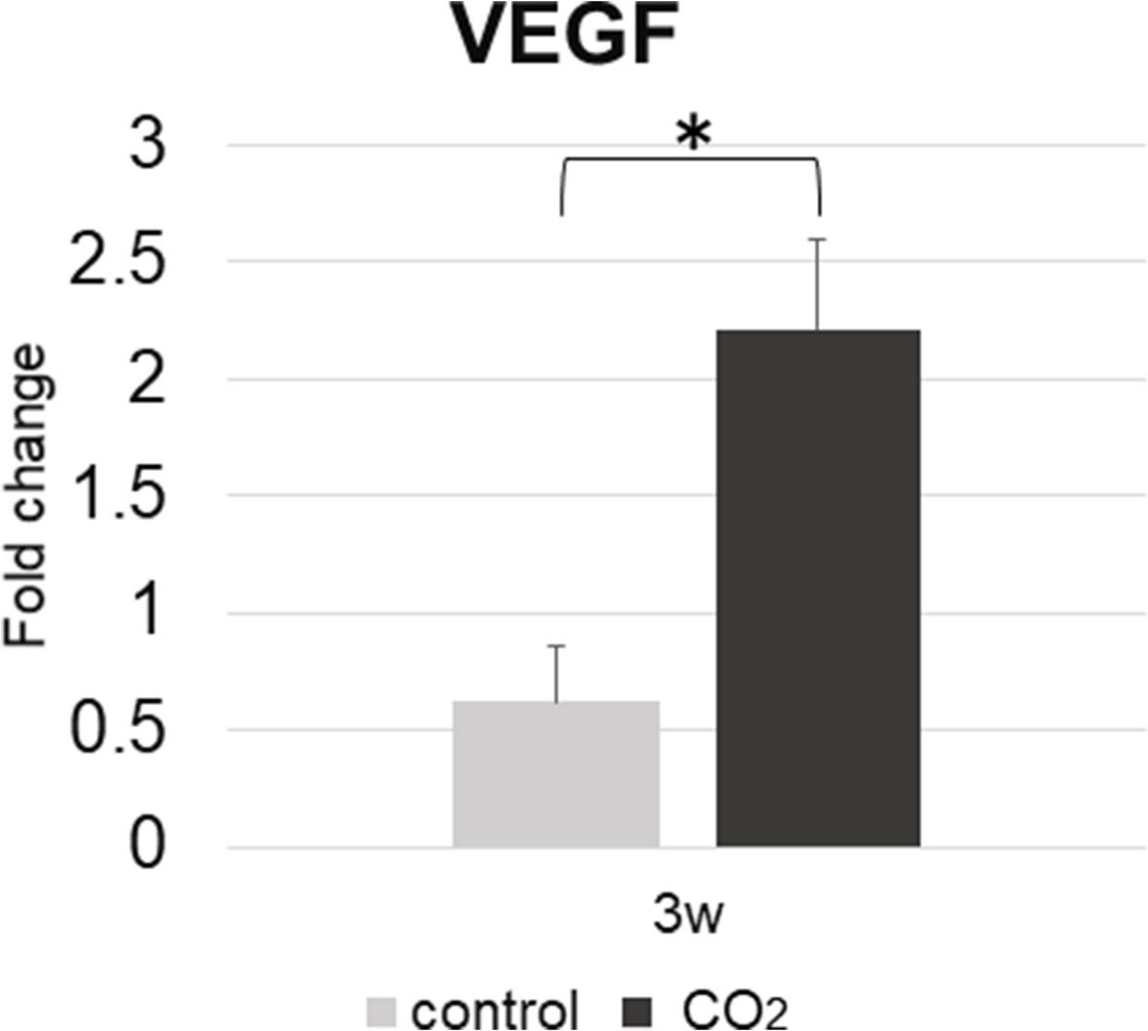
Expression level with standard error of VEGF of interest in each group at 3 weeks, which was measured by quantitative real-time PCR. (n = 6 in each group) (*p < 0.05 in the indicated group). VEGF, vascular endothelial growth factor

Real-time PCR was performed to evaluate the expression level of VEGF during bone healing. The gene expression of VEGF in the CO_2_ group was significantly higher than that in the control group at 3 weeks. (control group, 0.617 ± 0.240 vs. CO_2_ group, 2.213 ± 0.387) (Fig. 8) There were no significant differences between the groups at 1 and 2 weeks (Week 1: control group, 1.166 ± 0.130 vs. CO_2_ group, 3.335 ± 1.084; Week 2: control group, 2.313 ± 0.660 vs. CO_2_ group, 2.371 ± 0.558).

## Discussion

The current study demonstrated that topical cutaneous application of CO_2_ increased the bone healing rate in a rat femoral defect model at 4 weeks after surgery. Sato et al. [19] reported that bone healing in rat models with 1-, 2-, and 3-mm femoral defects was observed at 8 weeks postoperatively. As a preliminary experiment, we created 2 mm bone defect models in rat femurs and no specimens achieved bone healing. However, bone healing was achieved in all specimens when creating 1 mm bone defect models. In the present study, we initially examined bone healing at 4 weeks as too early a time point to attain bone healing using 1 mm bone defect models in rat femurs that are expected to accomplish bone union eventually. Although there was a significant difference in bone healing rate between CO_2_ group and control group at 4 weeks, all specimens of the two groups did not attain bone healing. We confirmed that all of the 1-mm bone defect models in the two groups reached bone healing at 6 and 8 weeks postoperatively. Therefore, it was considered that 4 weeks after surgery was an appropriate time point to investigate the effect of topical cutaneous application of CO_2_ on bone healing.

The histological assessment in this study demonstrated a significant acceleration of bone healing in the CO_2_ group at as early as 2 weeks postoperatively. This may suggest that topical cutaneous application of CO_2_ in bone defect promoted endochondral ossification which occurred in early stage of osteogenesis. Considering fracture healing, Koga et al. studied the effect of transcutaneous CO_2_ application using a rat fracture model and reported that the gene expression of VEGF, Osterix, and Runx2 increased in the callus of the CO_2_/hydrogel group, indicating that angiogenesis and bone formation were promoted [17]. Angiogenesis is an important factor of bone repair, and it has been shown to serve as a means for inflammatory cells, cartilage precursor cells, and bone precursor cells, which metabolically transport oxygen and nutrients, to reach the injured site [20–22]. It is supposed that angiogenesis plays an important role in bone formation in a bone defect, as well as in fracture healing, and that CO_2_ application may contribute to acceleration of bone formation via promotion of angiogenesis. This can be supported by the result of real-time PCR in the current study. The expression level of VEGF was significantly higher in the CO_2_ group compared to the control group at 3 weeks postoperatively, suggesting enhanced angiogenesis in the bone defect site.

On the other hand, considering the result of histological assessment, it seems that endochondral ossification occurred before angiogenesis, which might be interpreted as an incompatibility with the process of ordinary bone formation. However, the gene expression of VEGF increased in the CO_2_ group at 2 weeks. Although this was not statistically significant, it might reflect accelerated angiogenesis in the earlier phase than endochondral ossification by CO_2_ treatment. As for the significant upregulation of VEGF in the CO_2_ group at 3 weeks, the reasons could be speculated as follows. Three weeks is considered the later phase of endochondral ossification and the time at which angiogenesis into newly formed chondral tissue actively occurs. The CO_2_ application might affect this angiogenesis following endochondral ossification, leading to a significant increase of VEGF expression. The detailed relationship between CO_2_ application and VEGF is still unknown, and further investigation is necessary to elucidate that.

Our findings suggest that topical cutaneous application of CO_2_ could be a beneficial treatment for bone defect accelerating bone regeneration. In a clinical setting, our CO_2_ application system is minimally invasive and relatively less costly [17]. However, additional experiments are needed to investigate whether this system could achieve bone healing in larger animals for clinical application.

This study has one limitation. The change in CO_2_ concentration in the bone defect area during topical cutaneous application of CO_2_ was not measured directly. Previously, we demonstrated that CO_2_ application significantly lowered the intracellular pH of human muscle [15]. Koga et al. suggest that the change in pH at the fracture site caused by CO_2_/hydrogel treatment may be one of the mechanisms leading to the promotion of angiogenesis and blood flow [17]. Further investigation is required to clarify whether CO_2_ is actually absorbed into the tissue at the bone defect area and whether promotion of endochondral ossification is directly induced by the CO_2_ itself or by a secondary reaction to the topical cutaneous application of CO_2_.

## Conclusions

Topical cutaneous application of CO_2_ accelerated bone healing in a rat femoral defect model. Thus, CO_2_ application can be considered a novel and useful therapy for accelerating bone healing in bone defects, and further research on its efficacy in humans is warranted.

## Abbreviations

BMC: Bone mineral content
CO_2_: Carbon dioxide
EDTA: Ethylenediaminetetraacetic acid
GAPDH: Glyceraldehyde-3-phosphate dehydrogenase
PCR: Polymerase chain reaction
ROI: Region of interest
TMD: Tissue mineral density
TV: Total callus volume
vBMD: BMC/TV, volumetric bone mineral density
VEGF: Vascular endothelial growth factor
μ-CT: micro-computed tomography
